# Changes in Antiviral Prescribing for Children With Influenza in US Emergency Departments

**DOI:** 10.1001/jamanetworkopen.2025.38729

**Published:** 2025-10-22

**Authors:** Tess Stopczynski, Olla Hamdan, Justin Z. Amarin, James W. Antoon, Laura S. Stewart, Haya Hayek, James Chappell, Andrew J. Spieker, Eileen J. Klein, Janet A. Englund, Geoffrey A. Weinberg, Peter G. Szilagyi, John V. Williams, Marian G. Michaels, Julie A. Boom, Leila C. Sahni, Mary Allen Staat, Elizabeth P. Schlaudecker, Jennifer E. Schuster, Rangaraj Selvarangan, Ayzsa Tannis, Heidi L. Moline, Samantha M. Olson, Natasha B. Halasa

**Affiliations:** 1 Department of Biostatistics, Vanderbilt University Medical Center, Nashville, Tennessee; 2 Department of Pediatrics, Vanderbilt University Medical Center, Nashville, Tennessee; 3 Seattle Children’s Research Institute, Seattle, Washington; 4 University of Rochester School of Medicine and Dentistry & UR-Golisano Children’s Hospital, Rochester, New York; 5 Department of Pediatrics, University of California, Los Angeles, Los Angeles; 6 UPMC Children’s Hospital of Pittsburgh, University of Pittsburgh School of Medicine, Pittsburgh, Pennsylvania; 7 Department of Pediatrics, School of Medicine and Public Health, University of Wisconsin-Madison, Madison; 8 Texas Children’s Hospital and Baylor College of Medicine, Houston; 9 Cincinnati Children’s Hospital Medical Center, University of Cincinnati College of Medicine, Cincinnati, Ohio; 10 Department of Pediatrics, University of Missouri-Kansas City, Children’s Mercy Hospital, Kansas City; 11 Department of Pathology & Laboratory Medicine, University of Missouri Kansas City, Children’s Mercy Hospital, Kansas City; 12 Centers for Disease Control and Prevention, Atlanta, Georgia

## Abstract

**Importance:**

Despite national recommendations, antiviral prescribing in emergency departments (EDs) for children at higher risk of severe influenza, such as those younger than 5 years and those with specific underlying conditions, remains low.

**Objective:**

To assess whether there were changes in antiviral prescribing for children at higher risk of severe influenza in academic pediatric EDs before the COVID-19 pandemic (2016-2020) vs the late pandemic period (2021-2023).

**Design, Setting, and Participants:**

This multicenter, cross-sectional study included influenza-positive children younger than 18 years presenting to the ED at 1 of 7 US pediatric academic hospitals participating in the Centers for Disease Control and Prevention’s New Vaccine Surveillance Network. The analysis focused on children at higher risk of severe influenza seen in the ED from December 1, 2016, to June 30, 2023.

**Exposure:**

High risk of severe influenza.

**Main Outcomes and Measures:**

The primary outcome was antiviral prescribing. Children with influenza who met the criteria for higher risk of severe influenza were included. Antiviral prescribing practices were compared across the prepandemic and late pandemic periods. Mixed-effects logistic regression was used to identify factors associated with prescribing during the late pandemic period.

**Results:**

Of 3378 influenza-positive children (median [IQR] age, 3.9 [1.8-7.2] years), 2514 (74.4%; 1363 male [40.3%]) were classified as having higher risk of severe influenza during the prepandemic and late pandemic periods. Antiviral prescriptions decreased from 32.2% (622 of 1931 children) before the pandemic to 15.6% (91 of 583 children) in the late pandemic period, representing a 53% relative decrease. In the late pandemic period, symptom duration less than 2 days (adjusted odds ratio, 4.08; 95% CI, 2.49-6.71) and clinical influenza testing (adjusted odds ratio, 17.20; 95% CI, 4.08-72.37) were significantly associated with antiviral prescribing.

**Conclusions and Relevance:**

This multicenter, cross-sectional study of children with influenza in EDs found that, for children at higher risk of severe influenza illness, influenza antiviral prescribing decreased during the COVID-19 pandemic compared with prepandemic levels, despite unchanged treatment guidelines. Interventions are needed to support guideline-concordant prescribing in this population.

## Key Points

**Question:**

Did influenza antiviral prescribing for influenza-positive children at higher risk of severe illness in the emergency department (ED) change from 2016 to 2023, and what factors are associated with prescribing?

**Findings:**

In this cross-sectional study of 2514 children at higher risk for severe influenza in the ED, 32.2% received antiviral prescriptions before the COVID-19 pandemic (2016-2020) vs 15.6% in the late pandemic period (2021-2023). Antivirals were more commonly prescribed to children presenting within 2 days of symptom onset and among those who received clinical influenza testing.

**Meaning:**

These findings demonstrate a decline in prescribing despite unchanged treatment guidelines, highlighting opportunities to improve practice.

## Teaser

This cross-sectional study examines whether there were changes in antiviral prescribing for children at higher risk of severe influenza in academic pediatric emergency departments (EDs) before the COVID-19 pandemic (2016-2020) vs the late pandemic period (2021-2023).

## Introduction

Acute respiratory illness (ARI), particularly ARI caused by influenza, is a major reason for pediatric emergency department (ED) visits.^1^ Influenza can lead to severe illness and, in some cases, life-threatening complications, especially in young children and those with underlying medical conditions.^2^ Timely administration of antiviral medications has been shown to reduce both the severity and duration of influenza illness, thereby potentially decreasing risk of complications and need for hospitalization.^3,4,5^

Current recommendations by the American Academy of Pediatrics (AAP), Infectious Diseases Society of America (IDSA), and Centers for Disease Control and Prevention (CDC) state that hospitalized children and/or children with severe or prolonged illness and those at high risk for influenza complications, including those younger than 5 years or those with underlying medical conditions, should be treated as soon as possible with antivirals for suspected or confirmed influenza.^6,7^ Recent evidence suggests that approximately 30% of influenza-positive children at higher risk of severe influenza illness were prescribed antivirals during their ED visit in the years before the COVID-19 pandemic.^8^

The onset of the COVID-19 pandemic impacted health care practices, resulting in substantial changes in the ED workflows, resource allocation, and clinical decision-making.^9,10^ The widespread effects of the pandemic, along with the introduction of COVID-19 screening and treatment protocols, introduced new challenges and priorities within EDs, potentially affecting adherence to established influenza treatment guidelines. The considerable overlap in symptoms between COVID-19 and influenza further complicated clinical management, underscoring the need for accurate diagnostic testing to differentiate between them. Increased testing for both illnesses became an essential strategy to guide appropriate decisions, such as treatment, isolation, and investigation. Diagnostic uncertainty and intensified testing may have influenced physicians’ prescribing practices for influenza antivirals. However, it remains unclear whether the pandemic-related outcomes on the management of pediatric ARI in the ED led to changes in antiviral prescriptions for patients at increased risk of severe influenza.

Prompt and appropriate treatment of children at higher risk of severe influenza with antivirals can prevent severe outcomes, reduce burden on EDs, and broadly support public health.^11^ Therefore, the primary goal of this study was to use data from the CDC New Vaccine Surveillance Network (NVSN) over 7 influenza seasons before and after the onset of the COVID-19 pandemic to determine whether antiviral prescription for children at higher risk of severe influenza illness changed over time. In addition, we determined factors associated with influenza antiviral prescribing.

## Methods

### Study Population

This cross-sectional study included patients younger than 18 years enrolled between December 1, 2016, and June 30, 2023, in the ED at 1 of 7 NVSN surveillance sites: Cincinnati, Ohio; Houston, Texas; Kansas City, Missouri; Nashville, Tennessee; Pittsburgh, Pennsylvania; Rochester, New York; and Seattle, Washington. Between December 2016 and November 2019, 3 sites restricted enrollment in the ED to children younger than 5 years (Seattle, December 2016 to April 2017 and November 2017 to April 2018; Pittsburgh, December 2016 to April 2018; and Kansas City, December 2016 to April 2017 and November 2017 to April 2018). Children were deemed eligible for inclusion on the basis of previously described criteria.^12^ Eligible children had duration of ARI less than 14 days before enrollment and had fever and/or at least 1 other ARI; all symptoms were self-reported. Children were excluded from enrollment on the basis of study criteria including whether they experienced fever and neutropenia due to malignant neoplasm, were transferred from another hospital after an admission lasting more than 48 hours, were newborns who had never been discharged home, had been enrolled in this study within 14 days of their current visit or hospitalization, received influenza antivirals before the enrollment visit, or the status of their prior influenza antiviral receipt was unknown. We excluded children who received influenza antivirals before the ED visit to avoid confounding by prepresentation treatment on antiviral prescribing decisions made during their visit. The study was reviewed and approved by CDC and the institutional review boards at each of the 7 participating sites (45 CFR part 46.114; 21 CFR part 56.114). This study is reported in accordance with the Strengthening the Reporting of Observational Studies in Epidemiology (STROBE) reporting guideline for cross-sectional studies.

### Data and Specimen Collection

Research staff obtained written informed consent from the parent or guardian and assent from the child, if applicable, as a prerequisite to enrollment. The child’s parent or guardian was interviewed using a standardized case report form to collect sociodemographic and clinical information (ie, age, sex, race, ethnicity, symptoms, and duration of symptoms before enrollment). Race and ethnicity were self-reported or collected by medical record and are included in this study to describe patient demographics. Staff performed medical record reviews to record physical examination findings, underlying medical conditions, and clinical outcomes. The patient’s documentation included information on influenza antivirals (oseltamivir, zanamivir, baloxavir, and/or peramivir), either by administration in the ED or prescription at discharge.

Research respiratory specimens (eg, midturbinate nasal and/or oropharyngeal swabs) were collected from all patients at enrollment and were tested for influenza and respective subtypes via commercial or institution-specific in-house reverse transcription–polymerase chain reaction assays at each site. A previously collected respiratory specimen from practitioner-ordered clinical care was used when a research specimen was unobtainable.

### Influenza Season Definition

Study seasons were defined as the 12-month period from the first Sunday in July to the day before the first Sunday in July of the following calendar year. We defined the peak influenza season as the 13 consecutive weeks with the highest number of influenza cases for each site during their influenza season. We divided the study into 3 distinct time periods: prepandemic (December 1, 2016, to March 31, 2020), early pandemic (April 1, 2020, to June 30, 2021), and late pandemic (July 1, 2021, to June 30, 2023). Because of the small number of influenza-positive cases seen in the ED during the early pandemic period (8 cases), this period was excluded from analyses.

### Defining Children at Increased Risk of Severe Influenza

Influenza cases were identified as children who tested positive for influenza by either clinical or research testing methods. Clinical testing encompassed antigen-based and nucleic acid–based assays. Research testing results were not available to health care practitioners. Children were defined as being at higher risk of severe influenza illness according to AAP, IDSA, and CDC treatment recommendations for children with suspected or confirmed influenza.^6,7^ This includes children younger than 5 years and those with an underlying medical condition: respiratory disease, cardiovascular disease, kidney disease, hepatic disease, hematologic disease, metabolic disorder, neurologic disorder, or immunosuppression.^7^ Underlying medical conditions were self-reported or determined by medical record review. Statistical modeling included only children who were deemed to be at higher risk of severe influenza, although in the first paragraph of the Results section, we describe all influenza-positive children in the ED.

### Statistical Analysis

Descriptive statistics for demographic and clinical characteristics were reported as absolute and relative frequencies for categorical variables and as median (IQR) for continuous variables. Comparisons were made between influenza-positive individuals at higher risk of severe influenza illness by their antiviral prescription status and stratified by pandemic period (prepandemic or late pandemic). These comparisons were conducted using logistic regression for binary variables, multinomial logistic regression for multinomial variables, and linear regression for continuous variables, each including study site as a fixed effect and using cluster-robust SEs (ie, generalized estimating equations with a working independence structure) to account for clustering by individuals contributing more than 1 observation to the data. Our dataset included individuals who presented with influenza more than once (at any point during the study period) and were enrolled as separate encounters.

Mixed-effects logistic regression was used to evaluate the factors associated with antiviral prescription during the late pandemic period among children who were at higher risk of severe influenza illness. The model included variables selected a priori on the basis of discussions among coauthors regarding clinical factors likely to influence antiviral prescribing, informed by our prior research: age (years, modeled using restricted cubic splines with 3 knots), onset of symptoms longer than 2 days (yes or no), clinical influenza testing (yes or no), underlying medical condition (yes or no), influenza season (2021-2022 and 2022-2023), and peak influenza season (yes or no). Individual-level identifiers (accounting for repeat encounters) and study site were also included in the model as random effects.

We also fit a second mixed-effects logistic regression model to estimate the association between pandemic period and odds of antiviral prescribing. This model included all high-risk children across the study period and was structured similarly to the first model, with pandemic period (prepandemic vs late pandemic) included as the variable of interest. Owing to correlation with pandemic period, influenza season was excluded as a covariate from this model. A significance level of *P* < .05 was used for all analyses (2-tailed, where appropriate). Because of prespecification of analyses, we elected not to adjust for multiple comparisons; therefore, the family-wise type I error rate is higher than the nominal significance level of .05. We used R statistical software version 4.3.0 (R Project for Statistical Computing) for all calculations.

## Results

### Study Population

**Figure 1.**
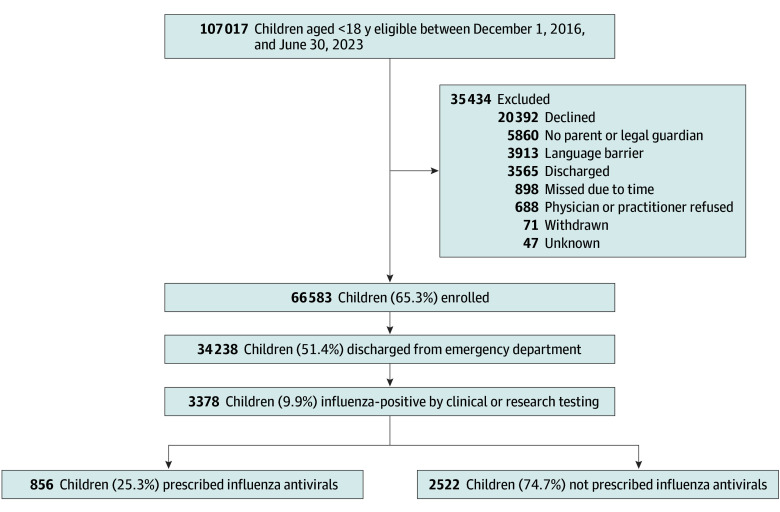
Flowchart of Study Participants Enrolled at 7 Children’s Hospitals, 2016-2023

Between December 1, 2016, to March 31, 2020, and July 1, 2021, to June 30, 2023, 34 238 children were seen in the ED and tested for influenza, of whom 3378 (9.9%) were influenza positive by clinical or research molecular testing (Figure 1). The median (IQR) age was 3.9 (1.8-7.2) years, and patients were predominantly male (1797 children [53.2%]). Overall, 856 influenza-positive children (25.3%) were prescribed an influenza antiviral. Oseltamivir was the most frequently prescribed antiviral (852 children [99.5%]) followed by baloxavir (4 children [0.5%]). There were 39 influenza-positive individuals who were enrolled in the study twice across the full surveillance period; all other observations were individuals with 1 enrollment of influenza.

### Antiviral Prescription Among Children at Higher Risk for Severe Influenza Illness

Of the 2514 influenza-positive children at higher risk of severe influenza illness during the prepandemic and late pandemic periods (1363 male [54.2%]), 772 (30.7%) were classified as higher risk of severe influenza by their underlying medical condition and 1742 (69.3%) were classified as higher risk because of age younger than 5 years. Among these children at high risk of severe influenza, 28.4% (713 of 2514 children) were prescribed an antiviral. When stratified by periods, 32.2% (622 of 1931 children) were prescribed an antiviral in the prepandemic period compared with 15.6% (91 of 583 children) in the late-pandemic period, representing a 53% relative decrease.

In the prepandemic period, children at higher risk of severe influenza illness who were prescribed antivirals had a similar observed median age and sex distribution compared with those who were not (Table). In addition, clinical influenza testing in the prepandemic period was significantly higher for children who were prescribed antivirals compared with those who were not (566 children [91.0%] vs 449 children [34.3%]), and a positive test was significantly associated with prescription of an antiviral (559 children [98.8%] vs 363 children [80.8%]). Those prescribed antivirals in the prepandemic period also were more likely to have visited the ED within the first 2 days of their symptoms compared with children not prescribed antivirals (361 children [58.0%] vs 456 children [34.8%]).

**Table.** Demographic and Clinical Characteristics of Influenza-Positive Children at Higher Risk of Severe Influenza Illness, 2016-2023^a^

Characteristic	Patients, No. (%)							
	Prepandemic period^b^				Late pandemic period^c^			
	Overall (n = 1931)	Antiviral prescription (n = 622)	No antiviral prescription (n = 1309)	*P* value	Overall (n = 583)	Antiviral prescription (n = 91)	No antiviral prescription (n = 492)	*P* value
Age, median (IQR), y	2.7 (1.3-4.3)	2.4 (1.1-4.5)	2.9 (1.4-4.3)	.94	3.3 (1.5-5.3)	3.9 (0.9-8.9)	3.2 (1.6-4.8)	.06
Sex								
Female	864 (44.7)	287 (46.1)	577 (44.1)	.39		42 (46.2)	245 (49.8)	.52
Male	1067 (55.3)	335 (53.9)	732 (55.9)		296 (50.8)	49 (53.8)	247 (50.2)	
Race and ethnicity, No./total No. (%)^d^								
Hispanic	497/1907 (26.1)	181/611 (29.6)	316/1296 (24.4)	.10	162/573 (28.3)	24/90 (26.7)	138/483 (28.6)	.69
Non-Hispanic Black	998/1907 (52.3)	300/611 (49.1)	698/1296 (53.9)		273/573 (47.6)	40/90 (44.4)	233/483 (48.2)	
Non-Hispanic White	263/1907 (13.8)	82/611 (13.4)	181/1296 (14.0)		89/573 (15.5)	16/90 (17.8)	73/483 (15.1)	
Non-Hispanic other	149/1907 (7.8)	48/611 (7.9)	101/1296 (7.8)		49/573 (8.6)	10/90 (11.1)	39/483 (8.1)	
Insurance status, No./total No. (%)^e^								
Private	222/1905 (11.7)	72/612 (11.8)	250/1293 (11.6)	.60	83/554 (15.0)	13/85 (15.3)	70/469 (14.9)	.12
Public	1541/1905 (80.9)	488/612 (79.7)	1053/1293 (81.4)		435/554 (78.5)	63/85 (74.1)	372/469 (79.3)	
Both	11/1905 (0.6)	5/612 (0.8)	6/1293 (0.5)		12/554 (2.2)	5/85 (5.9)	7/469 (1.5)	
Self-pay	131/1905 (6.9)	47/612 (7.7)	84/1293 (6.5)		24/554 (4.3)	4/85 (4.7)	20/469 (4.3)	
Clinical testing for influenza	1015 (52.6)	566 (91.0)	449 (34.3)	<.001	450 (77.2)	89 (97.8)	361 (73.4)	<.001
Influenza positive by clinical test, No./total No. (%)^f^	922/1015 (90.8)	559/566 (98.8)	363/449 (80.8)	<.001	426/450 (94.7)	86/89 (96.6)	340/361 (94.2)	.36
Symptom duration <2 d	817 (42.3)	361 (58.0)	456 (34.8)	<.001	195 (33.4)	56 (61.5)	139 (28.3)	<.001
Study site								
A	522 (27.0)	242 (38.9)	280 (21.4)	<.001	150 (25.7)	23 (25.3)	127 (25.8)	.008
B	184 (9.5)	7 (1.1)	177 (13.5)		28 (4.8)	0	28 (5.7)	
C	297 (15.4)	81 (13.0)	216 (16.5)		88 (15.1)	10 (11.0)	78 (15.9)	
D	183 (9.5)	95 (15.3)	88 (6.7)		46 (7.9)	6 (6.6)	40 (8.1)	
E	124 (6.4)	52 (8.4)	72 (5.5)		51 (8.7)	16 (17.6)	35 (7.1)	
F	385 (19.9)	108 (17.4)	277 (21.2)		127 (21.8)	18 (19.8)	109 (22.2)	
G	236 (12.2)	37 (5.9)	199 (15.2)		93 (16.0)	18 (19.8)	75 (15.2)	

^a^ Higher risk is defined as children younger than 5 years and/or those with an underlying medical condition (respiratory disease, cardiovascular disease, kidney disease, hepatic disease, hematologic disease, metabolic disorder, neurologic disorder, or immunosuppression).

^b^ The prepandemic period refers to December 1, 2016, to March 31, 2020.

^c^ The late pandemic period refers to July 1, 2021, to June 30, 2023.

^d^ Other included American Indian or Alaska Native, Asian, or Native Hawaiian or other Pacific Islander. Denominators differ from the overall total to indicate missingness by self-reported unknown.

^e^ Denominators differ from the overall total to indicate missingness by self-reported unknown.

^f^ Denominators differ from the overall total to indicate the number that were clinically tested for influenza.

Similar to the prepandemic period, children who were prescribed antivirals in the late pandemic period more frequently received clinical influenza testing (89 children [97.8%] vs 361 children [73.4%]) (Table), although for clinically tested patients, influenza detections did not differ between children who were prescribed antivirals vs those who were not. Notably, the percentage of children who were clinically tested for influenza was higher for both those who were prescribed antivirals and those who were not during the late pandemic compared with the prepandemic period (97.8% and 73.4% vs 91.0% and 34.3%, respectively). Consistent with the prepandemic period, those who presented to the ED within the first 2 days of symptoms during the late pandemic period were more likely to be prescribed antivirals compared with those who presented after 2 days (56 children [61.5%] vs 139 children [28.3%]).

**Figure 2.**
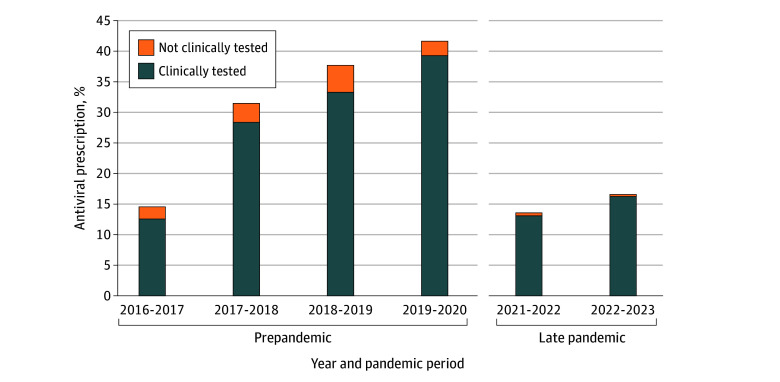
Percentage of Antiviral Prescriptions Among Influenza-Positive Children at Higher Risk of Severe Illness, 2016-2023 The year ranges overlap because influenza seasons are defined as the 12-month period from the first Sunday in July to the day before the first Sunday in July of the following calendar year.

Although influenza antiviral prescribing was lower in the late pandemic period compared with the prepandemic period, clinical influenza testing was higher among those who were influenza positive and at higher risk of severe illness (Table). Before the pandemic, 53.0% of the children (1310 of 2472 children) who were influenza positive and at increased risk of severe influenza were clinically tested for influenza, whereas in the late pandemic period, this increased to 77.6% (697 of 898 children). Furthermore, among those who tested positive for influenza at increased risk of severe influenza and were prescribed antivirals, the proportion of clinical testing was higher in the 2021 to 2022 and 2022 to 2023 influenza seasons, while the preceding seasonal proportions were slightly lower (Figure 2).

### Factors Associated With Antiviral Prescribing in the Late Pandemic Period

**Figure 3.**
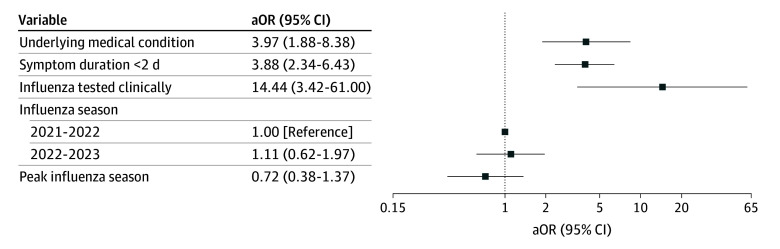
Adjusted Odds Ratios (aORs) of Antiviral Prescription Among High-Risk Children During the Pandemic Period A mixed-effects logistic regression model was used to compare the odds of antiviral prescription for each of the following variables (adjusting for all other variables in the model): age, symptom duration, underlying medical condition, clinical influenza testing (rapid antigen or polymerase chain reaction) before antiviral prescription, influenza season, peak influenza season, study site, and individual level identifier.

During the late pandemic period, antiviral prescriptions among 583 children at higher risk of severe influenza illness were associated with shorter symptom duration and clinical influenza testing. Children at higher risk who presented within 2 days of symptom onset had greater odds of being prescribed antivirals compared with higher risk children who presented more than 2 days after symptoms began (adjusted odds ratio [aOR], 4.08; 95% CI, 2.49-6.71) (Figure 3). Furthermore, children at higher risk who had clinical testing for influenza had greater odds of being prescribed antivirals (aOR, 17.20; 95% CI, 4.08-72.37). Although both spline terms for age were individually significant, a joint test of the age spline terms was not statistically significant.

### Antiviral Prescribing Across Pandemic Periods

The second mixed-effects logistic regression model indicated that antiviral prescribing was significantly lower in the late pandemic period compared with the prepandemic period (aOR, 0.17; 95% CI, 0.12-0.24) (eTable in Supplement 1). The direction and magnitude of associations for age, underlying conditions, symptom duration, and clinical influenza testing were consistent with those observed in the first model.

## Discussion

This cross-sectional study, spanning 7 influenza seasons from the multicenter NVSN, provides insights into changes in influenza antiviral prescription practices before and after the onset of the COVID-19 pandemic in the US. Among children at higher risk of severe influenza illness—all of whom are recommended to receive antiviral treatment for suspected or confirmed influenza^6,7^—we observed less-frequent antiviral prescription during the late pandemic influenza seasons compared with prepandemic seasons. Of the influenza-positive children at higher risk of severe influenza illness who were enrolled during the late pandemic period, 84.4% were not prescribed antivirals. These findings highlight that antiviral prescription management in the ED is lacking. Understanding reasons behind these trends is important to protect especially vulnerable pediatric patients from avoidable influenza morbidity. Prescribing rates had been increasing before the pandemic, which may reflect the impact of updated CDC and IDSA treatment guidelines published in 2018 and 2019, respectively; the relatively severe influenza seasons in all children in 2017 to 2018 and 2019 to 2020; and wider adoption of rapid molecular testing in EDs.

Overall, antiviral prescribing was lower among influenza-positive children at higher risk of severe illness during the late pandemic period compared with the prepandemic period. This observation aligns with CDC data documenting a substantial decline in antiviral treatment among hospitalized pediatric patients with influenza from 84.4% to 60.9% among children younger than 5 years and from 82.3% to 59.2% among those aged 5 to 17 years between the 2018 to 2019 and 2022 to 2023 seasons.^13,14,15^ Across all seasons, pediatric patients consistently received proportionately lower antiviral treatment compared with adults.^16^ These findings add to the concerning lack of antiviral prescribing in outpatient settings, where a previous study^17^ demonstrated that less than 40% of children younger than 2 years received guideline-concordant antiviral treatment before the pandemic. Our findings reveal a parallel trend of decreased antiviral prescribing in the ED setting for children at higher risk for severe influenza during the late pandemic and indicate that evolving patterns of influenza treatment among children at higher risk could be studied in all ambulatory care environments to observe whether similar trends exist elsewhere.

We suggest that several factors may explain the observed decline of antiviral prescriptions in the ED during the late pandemic period. The reduction in influenza antiviral prescriptions during the late pandemic period could be, in part, due to overlapping symptoms between influenza and COVID-19. Consequently, physicians may have been hesitant to prescribe influenza antivirals if they suspected an alternative cause of infection. A previous study^18^ conducted in Tennessee examining symptom presentation and SARS-CoV-2 infections demonstrated that ARI symptoms (runny nose, fever, sore throat, cough, and wheezing) were significantly associated with detection of SARS-CoV-2, although these are also common signs and symptoms of influenza. Thus, distinguishing between influenza and COVID-19 on the basis of symptoms alone may be challenging for physicians. Our findings emphasize the importance of reliable diagnostic tools to guide treatment decisions, particularly in the presence of multiple circulating respiratory viruses.

Qualitative studies by the CDC suggest a number of factors play a role in underprescribing influenza antivirals in the outpatient and ED settings, regardless of timing, including varying perceptions of antiviral effectiveness and adverse effects, differences in interpretation of influenza testing, and misunderstanding of the national societal guidelines.^19^ Concerns about oseltamivir’s adverse-effect profile may have influenced prescribing patterns. A meta-analysis found oseltamivir was associated with a modest (approximately 6%) increase in vomiting in children compared with placebo.^20^ Neuropsychiatric adverse effects were initially a postmarket concern; however, subsequent studies suggest that neuropsychiatric events are the result of underlying influenza illness, rather than oseltamivir treatment.^21,22,23,24,25,26^ However, practitioners and patients often cite concerns about vomiting or neuropsychiatric events when discussing oseltamivir, although a number of pediatric studies indicate that oseltamivir shortens illness, reduces transmission, and decreases the risk of hospitalization and complications.^20,27,28,29,30,31,32,33,34,35^ Misunderstanding of the effectiveness of influenza antivirals, or perceptions among practitioners and patients that influenza illness in children is not serious, may have impacted prescribing decisions.^19,36,37^ Finally, during the 2022 to 2023 respiratory viral season, the CDC received reports of limited generic oseltamivir availability in multiple locations across the US, which might have added a barrier to antiviral dispensing in some locations during that season.^38^

We found that clinical testing for influenza was more likely among children at higher risk of severe influenza during the late pandemic period compared with the prepandemic years. In addition, clinical testing was associated with antiviral prescribing. Among influenza-positive children at higher risk for severe influenza, more than one-half underwent clinical testing during the prepandemic period compared with more than three-quarters in the late pandemic period. This increase may reflect heightened awareness for respiratory infections and the need to differentiate influenza from COVID-19. We note that antiviral prescribing declined even as clinical testing increased, which suggests that if the denominator were limited to tested children, then the decline in treatment would be even more pronounced. Widening divergence between positive results of influenza diagnostic testing and administration of antiviral treatment across time periods raises questions about practitioner perspectives on the role of diagnostic assays in the therapeutic management of influenza infection among higher-risk children. This trend highlights the importance of continuously aligning laboratory diagnostic testing with clinical practices to maximize the impact of laboratory results on patient outcomes.

However, despite increased testing, guideline-concordant influenza antiviral prescribing remained low. Although AAP, IDSA, and CDC advise physicians not to delay antiviral treatment while waiting for influenza test results, some physicians may still rely on diagnostic testing to confirm infection, particularly in cases where the symptoms are not clearly distinguishable from those of COVID-19.^39^ According to organizational guidelines, even if a rapid influenza diagnostic test is negative, this result does not exclude influenza virus infection in patients with signs and symptoms suggestive of influenza; therefore, if clinically indicated, antiviral treatment should not be withheld and further molecular testing may be indicated.^40^

### Limitations

This study has limitations. First, our outcome was based on oseltamivir prescriptions written at discharge, and we did not have access to pharmacy fill records or medication adherence data. Therefore, we may have overestimated the proportion of children receiving the treatment as we cannot confirm all prescriptions are filled.^41^ Furthermore, we were unable to assess instances in which practitioners recommended antivirals but caregivers declined treatment, which may result in underestimation of practitioner adherence to guidelines. Second, some NVSN sites limited enrollment in the ED to children younger than 5 years during portions of the study, which may have excluded older children at higher risk of influenza. Third, NVSN criteria for eligibility changed during the study period; enrollment was restricted to children who had a duration of illness less than 10 days, although this modification was only restricted to a part of the 2022 to 2023 season. Fourth, variability in the duration and severity of influenza seasons from year to year and changes in resource availability may have influenced physicians’ prescribing behaviors across seasons. Other potentially unmeasured confounding factors affecting antiviral prescribing rates could include pandemic-related misinformation, ED workload, and factors that make patients less likely to fill prescriptions, such as out-of-pocket costs of antiviral medication and parental hesitancy owing to awareness of medication adverse effects.

## Conclusions

Among influenza-positive children at higher risk of severe illness, influenza antiviral prescription was lower in the period following the onset of the COVID-19 pandemic compared with the prepandemic period. However, the frequency of clinical testing among children at higher risk of severe influenza increased during the late pandemic period. This discrepancy between testing and treatment underscores the need to promote guideline-concordant antiviral prescribing, since timely initiation of treatment has been shown to reduce the severity and duration of influenza illness, thus lowering the risk of complications and hospitalization. Future efforts could explore factors contributing to underprescribing in the ED setting, including practitioner and patient knowledge, diagnostic decision-making, and medication access. Identifying and addressing such factors may help support more consistent use of antivirals among children at higher risk of severe influenza.

## Notes

Supplement 1. eTable. Adjusted odds ratios of antiviral prescription among high-risk children during the entire study period

Supplement 2. Data Sharing Statement

## References

[1] Rolfes MA, Foppa IM, Garg S, . Annual estimates of the burden of seasonal influenza in the United States: a tool for strengthening influenza surveillance and preparedness. Influenza Other Respir Viruses. 2018;12(1):132-137. doi:10.1111/irv.12486 29446233 PMC5818346

[2] Centers for Disease Control and Prevention. More than 100 flu-related deaths in children reported so far this season. March 8, 2024. Accessed November 18, 2024. https://www.cdc.gov/flu/whats-new/2023-2024-pediatric-flu-deaths.html?CDC_AAref_Val=https://www.cdc.gov/flu/spotlights/2023-2024/pediatric-flu-deaths.htm

[3] Campbell AP, Tokars JI, Reynolds S, . Influenza antiviral treatment and length of stay. Pediatrics. 2021;148(4):e2021050417. doi:10.1542/peds.2021-050417 34470815

[4] Walsh PS, Schnadower D, Zhang Y, Ramgopal S, Shah SS, Wilson PM. Association of early oseltamivir with improved outcomes in hospitalized children with influenza, 2007-2020. JAMA Pediatr. 2022;176(11):e223261. doi:10.1001/jamapediatrics.2022.3261 36121673 PMC9486642

[5] Wolf RM, Antoon JW. Influenza in children and adolescents: epidemiology, management, and prevention. Pediatr Rev. 2023;44(11):605-617. doi:10.1542/pir.2023-005962 37907421 PMC10676733

[6] Uyeki TM, Bernstein HH, Bradley JS, . Clinical practice guidelines by the Infectious Diseases Society of America: 2018 update on diagnosis, treatment, chemoprophylaxis, and institutional outbreak management of seasonal influenza. Clin Infect Dis. 2019;68(6):895-902. doi:10.1093/cid/ciy874 30834445 PMC6769232

[7] Committee on Infectious Diseases. Recommendations for prevention and control of influenza in children, 2023-2024. Pediatrics. 2023;152(4):e2023063773. doi:10.1542/peds.2023-063773 37641879

[8] Antoon JW, Hall M, Feinstein JA, . Guideline-concordant antiviral treatment in children at high risk for influenza complications. Clin Infect Dis. 2023;76(3):e1040-e1046. doi:10.1093/cid/ciac606 35867691 PMC10169402

[9] World Health Organization. Coronavirus disease (COVID-19) pandemic. Accessed November 18, 2024. https://www.who.int/europe/emergencies/situations/covid-19

[10] Stowell JR, Henry MB, Pugsley P, . Impact of the COVID-19 pandemic on emergency department encounters in a major metropolitan area. J Emerg Med. 2024;66(3):e383-e390. doi:10.1016/j.jemermed.2023.10.007 38278682

[11] Chotpitayasunondh T, Fischer TK, Heraud JM, . Influenza and COVID-19: what does co-existence mean? Influenza Other Respir Viruses. 2021;15(3):407-412. doi:10.1111/irv.12824 33128444 PMC8051702

[12] Perez A, Lively JY, Curns A, ; New Vaccine Surveillance Network Collaborators. Respiratory virus surveillance among children with acute respiratory illnesses—New Vaccine Surveillance Network, United States, 2016-2021. MMWR Morb Mortal Wkly Rep. 2022;71(40):1253-1259. doi:10.15585/mmwr.mm7140a1 36201373 PMC9541034

[13] Antoon JW, Amarin JZ, Hamdan O, . Antiviral use among children hospitalized with laboratory-confirmed influenza illness: a prospective, multicenter surveillance study. Clin Infect Dis. Published online December 17, 2024. doi:10.1093/cid/ciae573 39688383 PMC12497963

[14] Frutos AM, Ahmad HM, Ujamaa D, . Underutilization of influenza antiviral treatment among children and adolescents at higher risk for influenza-associated complications—United States, 2023-2024. MMWR Morb Mortal Wkly Rep. 2024;73(45):1022-1029. doi:10.15585/mmwr.mm7345a2 39541236 PMC11576051

[15] Stewart RJ, Flannery B, Chung JR, . Influenza antiviral prescribing for outpatients with an acute respiratory illness and at high risk for influenza-associated complications during 5 influenza seasons—United States, 2011-2016. Clin Infect Dis. 2018;66(7):1035-1041. doi:10.1093/cid/cix922 29069334 PMC6018951

[16] Naquin A, O’Halloran A, Ujamaa D, . Laboratory-confirmed influenza-associated hospitalizations among children and adults—Influenza Hospitalization Surveillance Network, United States, 2010-2023. MMWR Surveill Summ. 2024;73(6):1-18. doi:10.15585/mmwr.ss7706a1 39471107 PMC11537671

[17] Antoon JW, Sarker J, Abdelaziz A, . Trends in outpatient influenza antiviral use among children and adolescents in the United States. Pediatrics. 2023;152(6):e2023061960. doi:10.1542/peds.2023-061960 37953658 PMC10681853

[18] Biddle JE, Bonenfant G, Grijalva CG, . Association of symptoms and viral culture positivity for SARS-CoV-2-Tennessee, April-July 2020. Influenza Other Respir Viruses. 2024;18(6):e13318. doi:10.1111/irv.13318 39031815 PMC11190945

[19] Terrie YC. Tamiflu set to switch to OTC status. Pharmacy Times. December 2019. Accessed March 19, 2025. https://www.pharmacytimes.com/view/tamiflu-set-to-switch-to-otc-status

[20] Malosh RE, Martin ET, Heikkinen T, Brooks WA, Whitley RJ, Monto AS. Efficacy and safety of oseltamivir in children: systematic review and individual patient data meta-analysis of randomized controlled trials. Clin Infect Dis. 2018;66(10):1492-1500. doi:10.1093/cid/cix1040 29186364

[21] Harrington R, Adimadhyam S, Lee TA, Schumock GT, Antoon JW. The relationship between oseltamivir and suicide in pediatric patients. Ann Fam Med. 2018;16(2):145-148. doi:10.1370/afm.2183 29531106 PMC5847353

[22] Antoon JW, Williams DJ, Bruce J, . Population-based incidence of influenza-associated serious neuropsychiatric events in children and adolescents. JAMA Pediatr. 2023;177(9):967-969. doi:10.1001/jamapediatrics.2023.2304 37486679 PMC10366945

[23] Huh K, Kang M, Shin DH, Hong J, Jung J. Oseltamivir and the risk of neuropsychiatric events: a national, population-based study. Clin Infect Dis. 2020;71(9):e409-e414. doi:10.1093/cid/ciaa055 31996920

[24] Quertermous BP, Williams DJ, Bruce J, . Incidence of influenza-associated neurologic and psychiatric complications requiring hospitalization in children ages 5-17 years. Pediatr Infect Dis J. 2024;43(10):959-962. doi:10.1097/INF.0000000000004424 38869312 PMC11408088

[25] Casscells SW, Granger E, Kress AM, Linton A. The association between oseltamivir use and adverse neuropsychiatric outcomes among TRICARE beneficiaries, ages 1 through 21 years diagnosed with influenza. Int J Adolesc Med Health. 2009;21(1):79-89. doi:10.1515/IJAMH.2009.21.1.79 19526698

[26] Enger C, Nordstrom BL, Thakrar B, Sacks S, Rothman KJ. Health outcomes among patients receiving oseltamivir. Pharmacoepidemiol Drug Saf. 2004;13(4):227-237. doi:10.1002/pds.845 15255089

[27] Fry AM, Goswami D, Nahar K, . Effects of oseltamivir treatment of index patients with influenza on secondary household illness in an urban setting in Bangladesh: secondary analysis of a randomised, placebo-controlled trial. Lancet Infect Dis. 2015;15(6):654-662. doi:10.1016/S1473-3099(15)70041-1 25788164

[28] Hayden FG, Sugaya N, Hirotsu N, ; Baloxavir Marboxil Investigators Group. Baloxavir marboxil for uncomplicated influenza in adults and adolescents. N Engl J Med. 2018;379(10):913-923. doi:10.1056/NEJMoa1716197 30184455

[29] Ison MG, Portsmouth S, Yoshida Y, . Early treatment with baloxavir marboxil in high-risk adolescent and adult outpatients with uncomplicated influenza (CAPSTONE-2): a randomised, placebo-controlled, phase 3 trial. Lancet Infect Dis. 2020;20(10):1204-1214. doi:10.1016/S1473-3099(20)30004-9 32526195

[30] Monto AS, Kuhlbusch K, Bernasconi C, . Efficacy of baloxavir treatment in preventing transmission of influenza. N Engl J Med. 2025;392(16):1582-1593. doi:10.1056/NEJMoa2413156 40267424

[31] Liu JW, Lin SH, Wang LC, Chiu HY, Lee JA. Comparison of antiviral agents for seasonal influenza outcomes in healthy adults and children: a systematic review and network meta-analysis. JAMA Netw Open. 2021;4(8):e2119151. doi:10.1001/jamanetworkopen.2021.19151 34387680 PMC8363918

[32] Heinonen S, Silvennoinen H, Lehtinen P, . Early oseltamivir treatment of influenza in children 1-3 years of age: a randomized controlled trial. Clin Infect Dis. 2010;51(8):887-894. doi:10.1086/656408 20815736

[33] Lee JJ, Smith M, Bankhead C, . Oseltamivir and influenza-related complications in children: a retrospective cohort in primary care. Eur Respir J. 2020;56(5):1902246. doi:10.1183/13993003.02246-2019 32527739

[34] Venkatesan S, Myles PR, Leonardi-Bee J, . Impact of outpatient neuraminidase inhibitor treatment in patients infected with influenza A(H1N1)pdm09 at high risk of hospitalization: an individual participant data metaanalysis. Clin Infect Dis. 2017;64(10):1328-1334. doi:10.1093/cid/cix127 28199524 PMC5411393

[35] Antoon JW, Williams DJ, Bruce J, Sekmen M, Zhu Y, Grijalva CG. Influenza with and without oseltamivir treatment and neuropsychiatric events among children and adolescents. JAMA Neurol. Published online August 4, 2025. doi:10.1001/jamaneurol.2025.1995 40758339 PMC12322824

[36] Bassett HK, Rao S, Beck J, . Variability of clinician recommendations for oseltamivir in children hospitalized with influenza. Pediatrics. 2025;155(5):e2024069111. doi:10.1542/peds.2024-069111 40274268

[37] Pannaraj PS. Influenza antivirals in pediatrics: why aren’t we using all the available tools? Pediatrics. 2023;152(6):e2023063481. doi:10.1542/peds.2023-063481 37953646

[38] Kojima N, Peterson L, Hawkins R, Allen M, Flannery B, Uyeki TM. Influenza antiviral shortages reported by state and territorial public health officials, 2022-2023. JAMA. 2023;330(18):1793-1795. doi:10.1001/jama.2023.17244 37862007 PMC10646723

[39] Jones WA, Castro RC, Masters HL III, Carrico R. Influenza management during the COVID-19 pandemic: a review of recent innovations in antiviral therapy and relevance to primary care practice. Mayo Clin Proc Innov Qual Outcomes. 2021;5(6):974-991. doi:10.1016/j.mayocpiqo.2021.07.005 34414356 PMC8363430

[40] Centers for Disease Control and Prevention. Rapid influenza diagnostic tests. September 17, 2024. Accessed November 18, 2024. https://www.cdc.gov/flu/hcp/testing-methods/clinician_guidance_ridt.html

[41] Adams K, Garg S, Tartof SY, . Patterns in prescribing and dispensing of influenza antivirals among adults with influenza presenting to urgent care and emergency department settings, VISION Network, 2023-2024. Clin Infect Dis. Published online April 4, 2025. doi:10.1093/cid/ciaf178 40184184 PMC12353082

