# Supplementary material for: Changes in Antiviral Prescribing for Children With Influenza in US Emergency Departments
Source: JAMA Netw Open. 2025 Oct 22;8(10):e2538729. doi: 10.1001/jamanetworkopen.2025.38729 (PMC12547580; doi:10.1001/jamanetworkopen.2025.38729)
Supplement: Supplement 1. — eTable. Adjusted odds ratios of antiviral prescription among high-risk children during the entire study period [file jamanetwopen-e2538729-s001.pdf]

Supplemental Online Content

Stopczynski T, Hamdan O, Amarin JZ, et al. Changes in antiviral prescribing for children with influenza in US emergency departments. *JAMA Netw Open*. 2025;8(10):e2538729. doi:10.1001/jamanetworkopen.2025.38729

eTable. Adjusted odds ratios of antiviral prescription among high-risk children during the entire study period

This supplemental material has been provided by the authors to give readers additional information about their work.

© 2025 Stopczynski T et al. *JAMA Network Open*.

eTable. Adjusted odds ratios of antiviral prescription among high-risk children during the entire study period

	Estimate (95% CI)	p-value
RCS Age	0.69 (0.61, 0.78)	<0.001
RCS Age'	1.77 (1.45, 2.15)	<0.001
Underlying medical condition	1.84 (1.35, 2.50)	<0.001
Symptoms less than 2 days	2.82 (2.22, 3.59)	<0.001
Flu tested clinically	20.97 (13.97, 31.48)	<0.001
Peak flu season	1.11 (0.80, 1.52)	0.54
Late pandemic period	0.17 (0.12, 0.24)	<0.001

© 2025 Stopczynski T et al. *JAMA Network Open*.
