# Supplementary material for: Changes in Antiviral Prescribing for Children With Influenza in US Emergency Departments
Source: JAMA Netw Open. 2025 Oct 22;8(10):e2538729. doi: 10.1001/jamanetworkopen.2025.38729 (PMC12547580; doi:10.1001/jamanetworkopen.2025.38729)
Supplement: Supplement 2. — Data Sharing Statement [file jamanetwopen-e2538729-s002.pdf]

Data Sharing Statement

Stopczynski. Changes in Antiviral Prescribing for Children With Influenza in US Emergency Departments. *JAMA Netw Open*. Published October 22, 2025.
doi:10.1001/jamanetworkopen.2025.38729

Data
Data available: No
